# A New Calibration Method Using Low Cost MEM IMUs to Verify the Performance of UAV-Borne MMS Payloads

**DOI:** 10.3390/s150306560

**Published:** 2015-03-19

**Authors:** Kai-Wei Chiang, Meng-Lun Tsai, El-Sheimy Naser, Ayman Habib, Chien-Hsun Chu

**Affiliations:** 1 Department of Geomatics, National Cheng-Kung University, No.1, Daxue Rd., Tainan 70101, Taiwan; E-Mails: kwchiang@mail.ncku.edu.tw (K.-W.C.); taurus.bryant@msa.hinet.net (M.-L.T.); 2 Department of Geomatics Engineering, University of Calgary, Calgary City, AB T2N 1N4, Canada; E-Mail: elsheimy@ucalgary.ca; 3 Lyles School of Civil Engineering, Purdue University, Lafayette city, IN 47907, USA; E-Mail: ahabib@purdue.edu

**Keywords:** UAV, IMU, GPS, MMS, DG, calibration

## Abstract

Spatial information plays a critical role in remote sensing and mapping applications such as environment surveying and disaster monitoring. An Unmanned Aerial Vehicle (UAV)-borne mobile mapping system (MMS) can accomplish rapid spatial information acquisition under limited sky conditions with better mobility and flexibility than other means. This study proposes a long endurance Direct Geo-referencing (DG)-based fixed-wing UAV photogrammetric platform and two DG modules that each use different commercial Micro-Electro Mechanical Systems’ (MEMS) tactical grade Inertial Measurement Units (IMUs). Furthermore, this study develops a novel kinematic calibration method which includes lever arms, boresight angles and camera shutter delay to improve positioning accuracy. The new calibration method is then compared with the traditional calibration approach. The results show that the accuracy of the DG can be significantly improved by flying at a lower altitude using the new higher specification hardware. The new proposed method improves the accuracy of DG by about 20%. The preliminary results show that two-dimensional (2D) horizontal DG positioning accuracy is around 5.8 m at a flight height of 300 m using the newly designed tactical grade integrated Positioning and Orientation System (POS). The positioning accuracy in three-dimensions (3D) is less than 8 m.

## 1. Introduction

Mobile mapping is executed by producing more than one image that includes the same object acquired from different positions, allowing the 3D positions of the object with respect to the mapping frame to be measured [1]. Multi-sensors can be mounted on a variety of platforms, such as satellites, aircraft, helicopters, terrestrial vehicles, water-based vessels, and even people. As a result, mapping has become mobile and dynamic. Mobile mapping technology enables DG by integrating GPS and INS, which makes Exterior Orientation Parameters (EOPs) of accurate images available at any given time [2]. The integration of INS and GPS improves the geo-referencing of photogrammetric data and frees it from operational restrictions that require ground reference information. Operational flexibility is greatly enhanced in all cases where a block structure is not needed [3]. Costs are considerably reduced, especially in areas where little or no ground control is available [4].

As the number of natural disasters caused by climate change increases, rapid spatial information acquisition capability using remote sensing and mobile mapping applications has received wide attention. Consequently, the development of a rapidly deployable and low cost system for collecting near-real-time spatial information without any ground reference over the disaster area should be of great interest. 

The current achievable accuracy of commercial airborne MMSs is sufficient for numerous mapping applications. In addition, cost has decreased and production efficiency has increased with the use of DG-based photogrammetric platforms [4]. An airborne MMS that is relatively free of government regulations and inexpensive but maintains high mobility for small area surveys or rapid spatial information acquisition is desirable for urgent response events such as disaster relief and assessment.

Satellite images can be constrained by a number of factors, such as weather, availability of stereo coverage, temporal and geometric resolution, minimum area order and price. Thus airborne platforms such as aircraft, helicopters, kites, balloons and UAVs are good and generally cheap alternatives, especially since recent developments in small and medium format digital cameras have made great advances in automated image processing. Numerous studies have been conducted for applying UAV to photogrammetric research [1,5,6]. Nowadays despite the widespead availability of very high resolution satellite imagery, large scale photogrammetric mapping applications still primarily use aerial images because for large areas, aircraft are usually employed as a platform for acquiring aerial images. However, for small and remote area mapping, UAV is a very good and inexpensive platform and imaging alternative. It should be particularly attractive for developing countries. 

Generally speaking, the main applications of UAVs may be defined as observation, maintenance, surveillance, monitoring, remote sensing and security tasks [7]. In recent years, more and more UAV-based photogrammetric platforms have been developed and their performance has been proven in certain scenarios [8]. Chiang *et al.* [9] developed a DG based UAV photogrammetric platform where an INS/GPS integrated POS system was implemented to provide the DG capability of the platform. The preliminary results show horizontal DG positioning accuracies in the East and North directions of below 10 m at a flight height of 300 m without using any GCP. The positioning accuracy in the Up direction is less than 15 m. Such accuracy is good enough for near real time disaster relief.

Rehak *et al.* [10] developed a low cost UAV for direct geo-referencing. The advantage of such a system lies in its high maneuverability, operation flexibility as well as capability to acquire image data without the need of establishing GCPs. Moreover, the precise geo-referencing has better final mapping accuracy when employing integrated sensor orientation, limiting the number and distribution of GCPs, thus saving time in their signalization and surveying.

Generally speaking, the selection of a platform is application dependent. The primary objective of developing a UAV based photogrammetric platform is to meet requirements such as small operational area, rapid deployment, low cost, high mobility, and acceptable positioning accuracy. Therefore, it is not practical to use such platforms as replacements for conventional photogrammetric applications [11].

## 2. Problem Statement 

**Figure 1:**
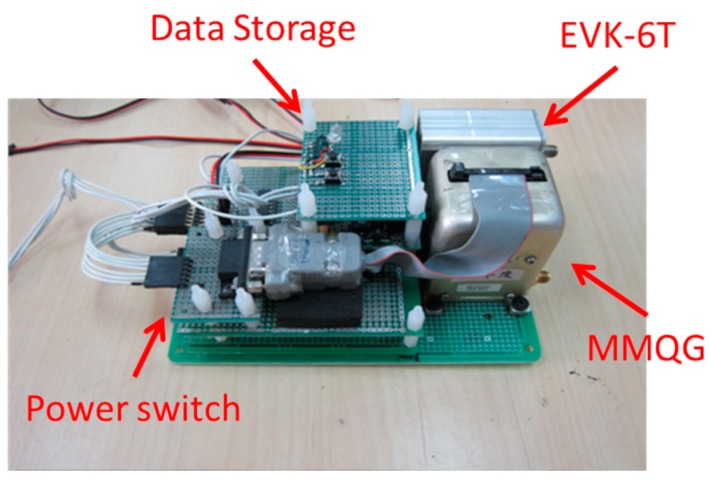
The DG module configuration.

As indicated previously, Chiang *et al.* [9] utilized a low cost INS/GPS integrated POS system to provide the DG capability of the UAV photogrammetric platform. Figure 1 shows a DG module proposed for facilitating GCP free photogrammetry applications and INS/GPS integrated POS aided bundle adjustment photogrammetry. The EVK-6T GPS receiver from U-blox is used in the DG module. This model was chosen because of its L1 carrier phase measurements for DGPS processing, which provides sufficient positioning accuracy. 

The IMU used for the DG module is an MMQ-G from BEI SDID (Concord, CA, USA). This model has been chosen due to its compact size and weight. The MMQ-G IMU integrates MEMS quartz rate sensors (100 deg/h in run bias) and vibrating quartz accelerometers. The total budget of the proposed POS module is around 10,000 US dollars. MEMS inertial sensors have advanced rapidly; thus, the inclusion of MEMS inertial sensors for UAV-borne MMS applications has good potential in terms of cost and accuracy.

With the advance of MEMS inertial technology, some commercial MEMS IMUs now provide better sensor stability while significantly reducing POS cost. Therefore, the first objective of this study was to develop a new POS module using a tactical grade IMU with 6 deg/h gyro in run bias but costing only one third of the POS module proposed in [9]. 

**Figure 2:**
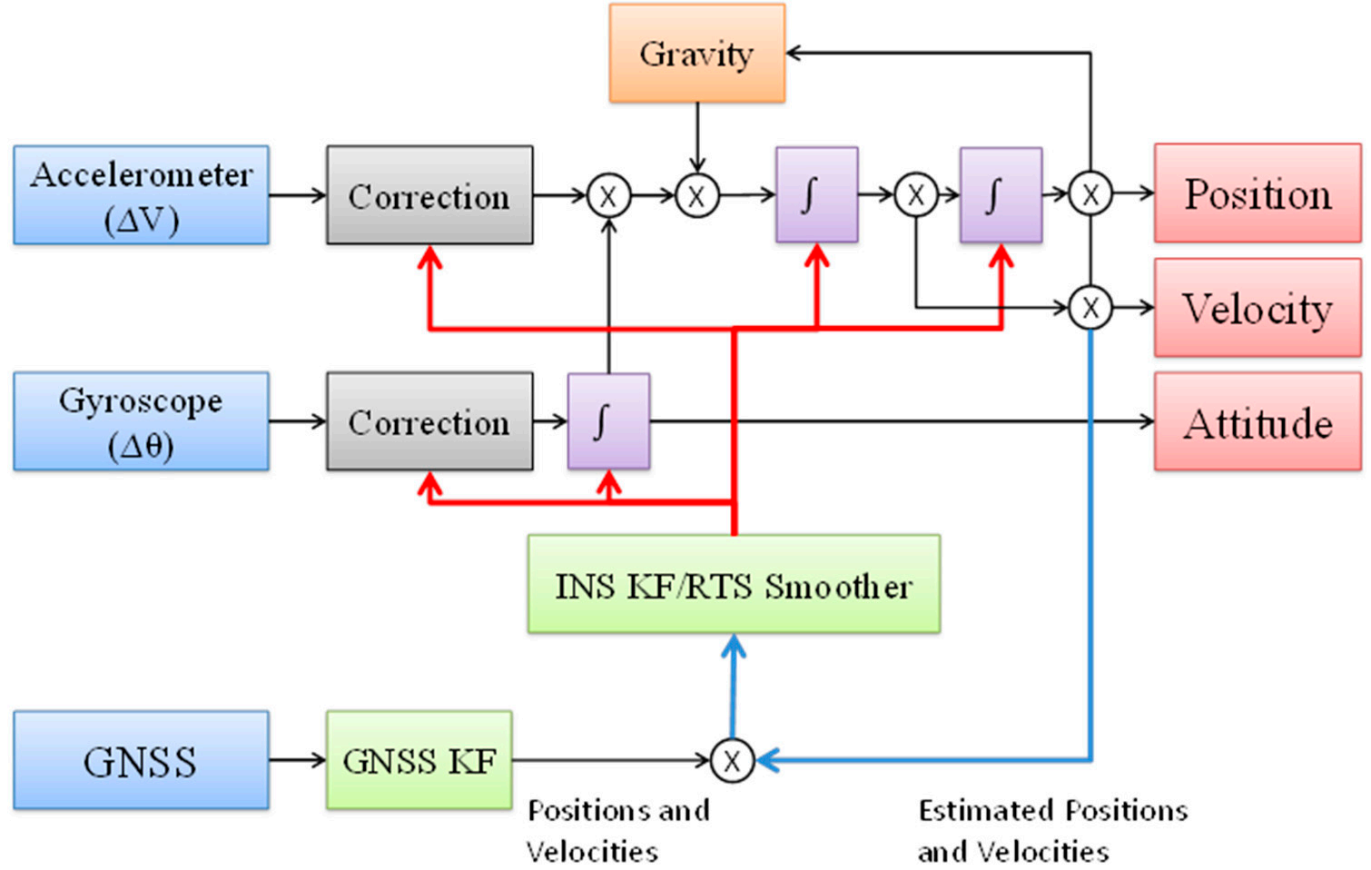
The LC integration scheme.

The most common integration scheme used today is the Loosely Coupled (LC) integration scheme, as shown in Figure 2. The position and velocity estimated by the GPS Kalman filter (KF) are processed in the navigation KF to aid the INS, a process also known as decentralized, or cascaded, filtering. This kind of integration has the benefit of a simpler architecture that is easy to utilize in navigation systems. However, the errors in the position and velocity information provided by the GPS KF are time-correlated, which can cause degradation in performance or even instability of the navigation KF if these correlations are not compensated for [12].

In the case of incomplete constellations, *i.e.*, fewer than four satellites in view, the output of the GPS receiver has to be ignored completely, leaving the INS unaided [13]. When a UAV flies in the open sky, the GPS signals are not obstructed or reflected by high buildings. There is no GPS outage and the user can receive data from more than four satellites, theoretically. 

However, the vibration of the UAV platform and certain maneuvers, such as sharp turns or sudden movements due to strong winds, can cause a loss of the logged GPS raw measurements [14].

This problem grows worse when carrier phase measurements are applied. Thus the accuracy of the POS solutions deteriorates significantly when a low cost MEMS IMU and the LC scheme are used during partial GPS outages. Therefore the second objective of this study is to apply a robust INS/GPS integration scheme to avoid the partial GPS outages taking place in UAV scenarios. 

**Figure 3:**
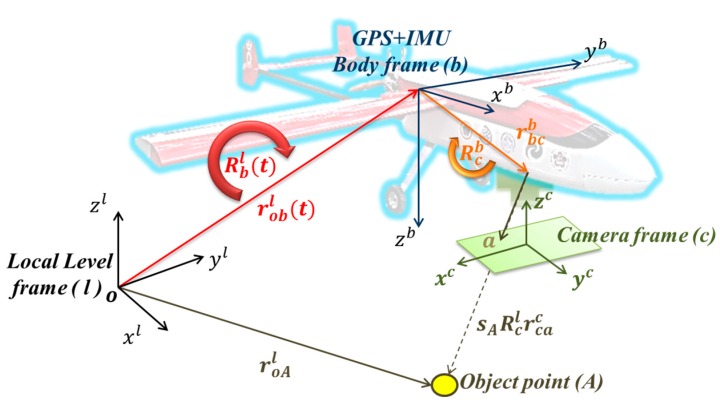
The concept of airborne DG.

Figure 3 and Equation (1) illustrate the general concept of the airborne DG. With this implementation, the coordinates of a mapping feature can be obtained directly through measured image coordinates. 

This procedure works based on *a priori* knowledge of various systematic parameters, as shown in the following representation:

(1) $${\text{r}}_{\text{oA}}^{\text{l}}={\text{r}}_{\text{ob}}^{\text{l}}(\text{t})+{\text{R}}_{\text{b}}^{\text{l}}(t)\left({\text{s}}_{A}{\text{R}}_{\text{c}}^{\text{b}}{\text{r}}_{\text{ca}}^{\text{c}}+{\text{r}}_{\text{bc}}^{\text{b}}\right)$$

In the formula, the “r” means a vector and “R” means a rotation matrix. Their superscripts and subscripts represent the frame. But the subscript of vector means start-point and end-point of this vector. ${\text{r}}_{oA}^{\text{l}}$ is the coordinate vector of feature point (A) in the Local Level frame (LLF, l-frame); ${\text{r}}_{\text{ob}}^{l}(t)$ is the interpolated coordinate vector of the navigation sensors (INS/GPS) in the l-frame; $s_{A}$ is a scale factor, determined by stereo techniques, laser scanners or a Digital Terrain Model (DTM);${\text{ R}}_{\text{b}}^{l}(t)$ is the interpolated rotation matrix from the navigation sensor body frame (b-frame) to the l-frame; (t) is the time of exposure, *i.e.*, the time of capturing the images, determined by synchronization; ${\text{R}}_{\text{c}}^{\text{b}}$ is the rotation matrix from the camera frame (c-frame) to the b-frame, determined by calibration; ${\text{r}}_{\text{ca}}^{\text{c}}$ is the coordinate vector of the point (a) in the c-frame (*i.e*., image coordinate); and ${\text{r}}_{\text{bc}}^{b}$ is the vector between the IMU center and the camera perspective center in the b-frame, determined by calibration.

**Figure 4:**
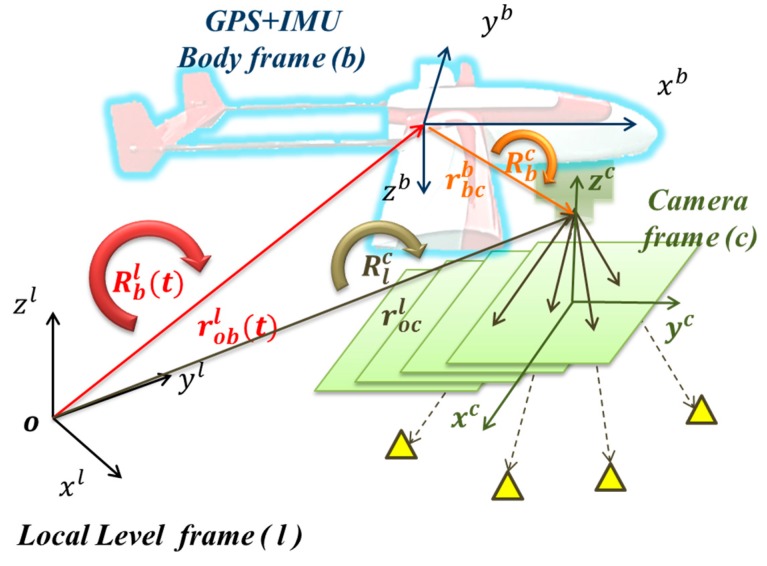
Concept of boresight angle calibration.

**Figure 5:**
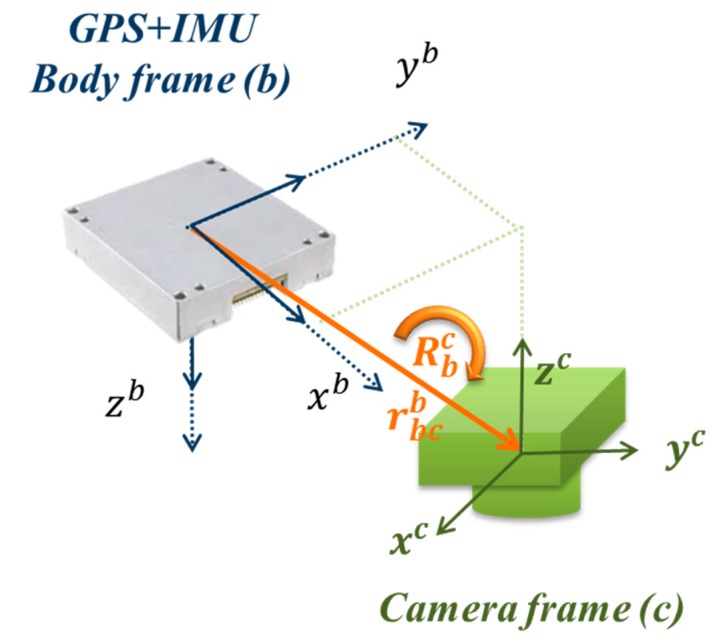
Concept of lever arm calibration.

The physical meanings of ${\text{R}}_{\text{c}}^{\text{b}}$ and $r_{bc}^{\text{c}}$ are given in Figure 4 and Figure 5, respectively. Traditional calibration procedure is implemented to acquire the rotation matrix (${\text{R}}_{\text{c}}^{\text{b}}$) between the camera and IMU by using the rotation matrix (${\text{R}}_{\text{b}}^{l}$) provided by the IMU and the rotation matrix $\left({\text{R}}_{\text{c}}^{l}\right)$ provided by conventional bundle adjustment using the l-frame’s control field during the calibration procedure using the following equation [15]:

(2) $${\text{R}}_{\text{c}}^{\text{b}}={\text{R}}_{\text{l}}^{\text{b}}{\text{R}}_{\text{c}}^{\text{l}}$$

The lever arm vector $r_{GPSb}^{b}$ between the GPS phase center and the IMU center is determined through a surveying process. The lever arm vector $r_{bc}^{b}$ between the camera and the IMU centers is determined through a two-step procedure: first, the EOPs of the images are calculated through bundle adjustment by measuring the image points when the flight mission had completed, and second, the interpolation of INS/GPS smoothed POS solutions at the image exposure time is implemented. The lever arm and boresight angle are obtained by comparing the differences of the position and the attitude between the EOP and the interpolated INS/GPS solutions using the following equation:

(3) $${\text{r}}_{\text{bc}}^{\text{b}}={\text{R}}_{\text{l}}^{\text{b}}\left(\begin{array}{c} {\text{X}}_{\text{oc}}^{\text{l}}-{\text{X}}_{\text{ob}}^{\text{l}}\\ {\text{Y}}_{\text{oc}}^{\text{l}}-{\text{Y}}_{\text{ob}}^{\text{l}}\\ {\text{Z}}_{\text{oc}}^{\text{l}}-{\text{Z}}_{\text{ob}}^{\text{l}} \end{array}\right)$$

where $r_{bc}^{b}$ is the lever arm vector to be estimated, $({\text{X}}_{\text{ob}}^{\text{l}}$, ${\text{Y}}_{\text{ob}}^{\text{l}}$,${\text{ Z}}_{\text{ob}}^{\text{l}})$ represents the positional vector of the INS center in the l-frame provided by INS/GPS integrated POS solutions, and $({\text{X}}_{\text{oc}}^{\text{l}}$, ${\text{Y}}_{\text{oc}}^{\text{l}}$, ${\text{ Z}}_{\text{oc}}^{\text{l}})$ represents the positional vector of the camera center in the l-frame provided by bundle adjustment. Once these parameters are well calibrated and the sensors are fixed on the platform, the proposed platform will be able to conduct GCP-free DG missions without conventional bundle adjustments for future flights.

However, in addition to those lever arms and boresight angles, the camera shutter delay that represents the time delay between the trigger time used for interpolating POS solutions and the exposure time when an image is taken should be calibrated simultaneously in kinematic mode, as explained in [9]. In practice, the trigger signal is sent to the camera (to take a picture) and to the recorder (to record the time mark) simultaneously. After this, the smoothed INS/GPS solutions can be interpolated at the time mark of each image. However, the camera exposure time will always be slightly different from the recorded time mark due to the delay caused by signal transmission time. This deviation of time leads to a systemic shift of position and attitude of each image along the forward direction. Therefore, exposure time delay compensation should be applied to estimate the magnitude of the time delay at each exposure station. To develop a system to compensate for this situation, the third objective of this study is to produce a new calibration method to solve this problem. The proposed method not only estimates the lever-arm and boresight, but estimates the deviation of time using the same measurements used by the traditional calibration method.

## 3. The Configuration of the Proposed Platform

**Figure 6:**
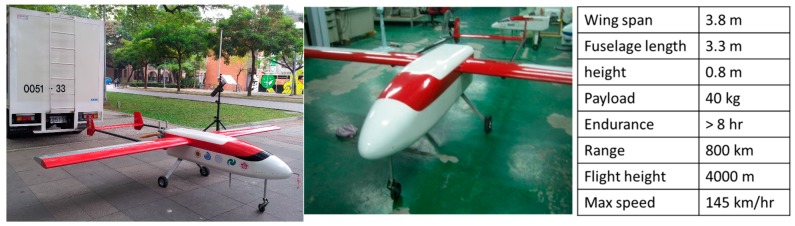
The proposed UAV platform.

**Figure 7:**
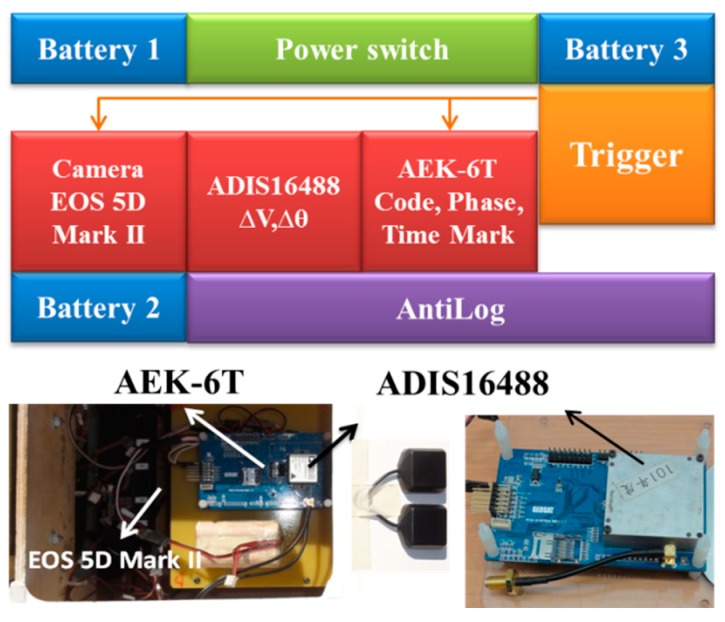
The configuration of DG module.

The proposed UAV platform and its specifications are illustrated in Figure 6, in which it can be seen that the proposed UAV platform is designed for medium range applications. The wing span is 4 m and the payload is 40 kg. The flexible flight altitude and eight–hour maximum flight-time make the platform suitable for small area and large scale photogrammetric missions. This model is jointly developed by the Department of Geomatics, NCKU and GeoSat Informatics Technology Co. Figure 7 depicts the tactical grade DG module designed in this study to facilitate direct photogrammetry as well as INS/GPS POS aided bundle adjustment.

**Figure 8:**
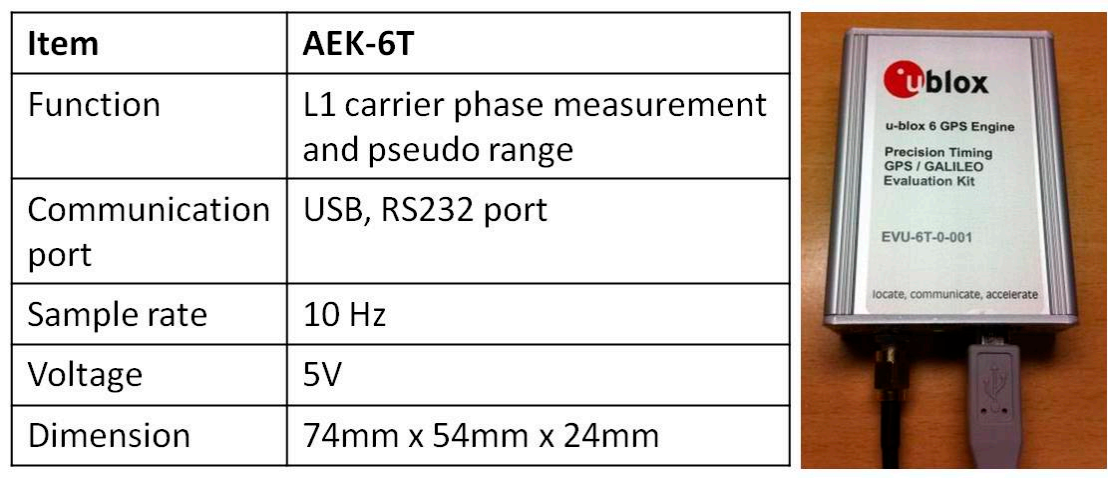
The GPS receiver of DG module.

Figure 8 illustrates the specifications of the GPS receiver, AEK-6T (Thalwil city, Switzerland) from the U-blox, which is applied in the DG module. This model has been chosen because it can provide L1 carrier phase raw measurements that can be applied for differential GPS processing with single frequency carrier phase measurements to provide sufficient positioning accuracy. In addition, it supplies Pulse Per Second (PPS) output used to synchronize the time mark used to trigger the DG module’s camera.

**Figure 9:**
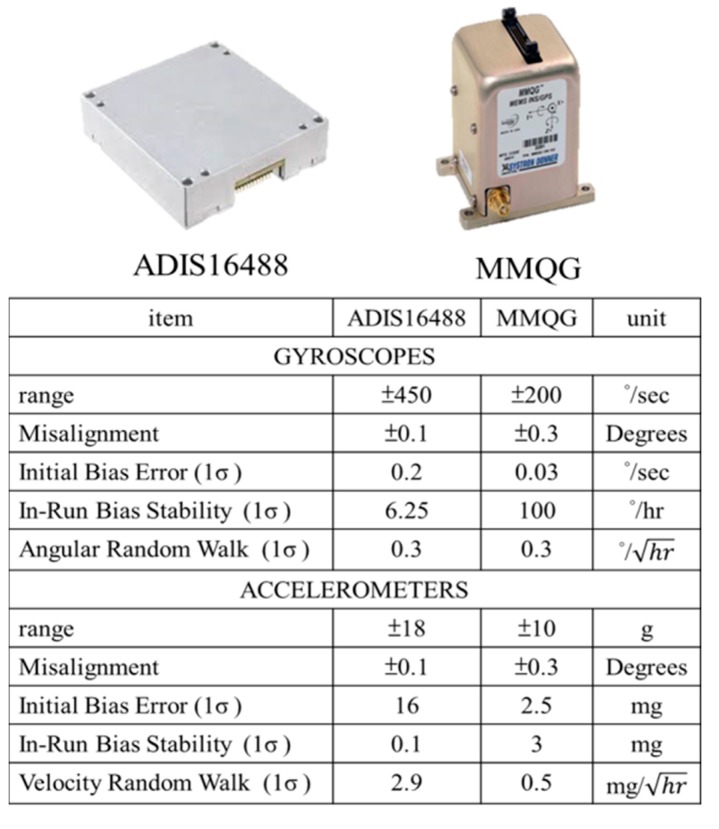
The IMUs for DG module.

**Figure 10:**
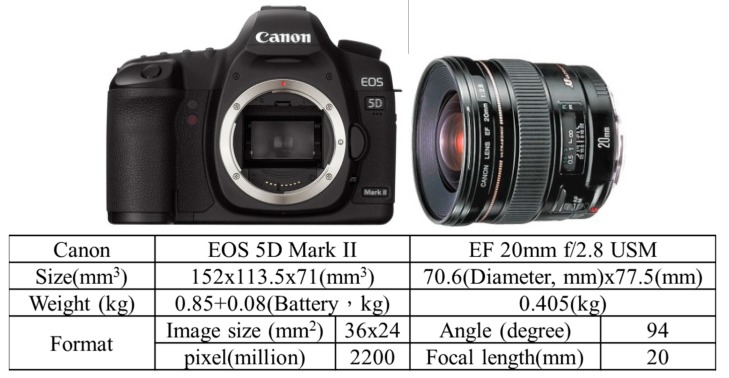
Canon EOS 5D Mark II & EF 20 mm f/2.8 USM.

Figure 9 illustrates the two IMUs used for the previous and new DG module, MMQ-G from BEI SDID and ADIS16488 (Newburyport city, MA, USA) from Analog Devices, respectively. These models have been chosen because of their compact size and weight. The retail price of the ADIS16488 IMU was around 1500 USD while the MMQ-G was 10,000~12,000 USD in 2008. Based on the specifications given below, the new version of the DG module is at least six times superior to previous version in terms of the quality of inertial sensors, but costs only one fifth of the original budget. A digital camera (EOS 5D Mark II, Canon, Tokyo city, Japan) is applied in this study. Figure 10 shows the picture and specifications of the camera. 

To supply the power required for the individual sensors with various power requirements from the battery, a power switch module has been designed. An RS232 port is used to transmit the measurements collected by the MMQ-G/ADIS16488 IMU to the data storage module. Since the camera has its own power supply, it is not considered in the power supply design. The pixel size of camera is 0.0064 mm and the focal length is fixed on about 20 mm. The data storage module used to record the measurements collected by MMQ-G/ADIS16488, EVK-6T, and the synchronized time mark used to trigger the camera is an Antilog from Martelec (Alton city, UK). It was chosen due to its flexibility, low power consumption, and reliability. Since the camera has its own storage mechanization, it is not included in this module.

## 4. Proposed Calibration Algorithm

As mentioned previously, the lever arms and boresight angles can be obtained by a traditional calibration method. When calibrating the lever arms and boresight angles, the perspective center of each image ($r_{oc}^{l}$) is exactly known after executing the bundle adjustment; the calculation of the INS/GNSS position vector ($r_{ob}^{l}$) and rotation matrix ($R_{l}^{b}$) is conducted by the interpolation at the trigger time received, after which the lever arms ($r_{bc}^{b}$) can be solved using the following equation:

(4) $$r_{bc}^{b}(t)=R_{l}^{b}(t)(r_{oc}^{l}\left(t\right)-r_{ob}^{l}\left(t\right))$$

In terms of the boresight angles’ calibration, the rotation matrix between the camera frame and the local level frame of each image ($R_{c}^{l}$) is also obtained through the bundle adjustment results, and the rotation matrix between the body frame and mapping frame of each image can be obtained through integrated solutions. The rotation matrix ($R_{c}^{b}$) can be calculated using the matrix multiplication:

(5) $$R_{c}^{b}(t)=R_{l}^{b}(t)R_{c}^{l}(t)$$

However, there is another important parameter: exposure time delay (∆t). This is the time difference between the timing recorded and the actual camera exposure. The traditional calibration formula supposes that time delay is zero. Because $R_{c}^{l}(t)$ in the formula is solved from picture through photogrammetry, the “t” should be the exposure time of camera. To compensate for this gap, the DG equation has been modified as follows:

(6) $$t_{c}=t_{b}+∆t$$

(7) $$r_{oA}^{l}=r_{ob}^{l}\left(t_{c}\right)+R_{b}^{l}\left(t_{c}\right)\left(r_{bc}^{b}+sR_{c}^{b}r_{ca}^{c}\right)\;=r_{ob}^{l}\left(t_{b}+\Delta t\right)+R_{b}^{l}(t_{b}+\Delta t)(r_{bc}^{b}+sR_{c}^{b}r_{ca}^{c})$$

where, “$t_{c}$” is the exposure time of the camera and “$t_{b}$” is the time recorded by the recorder. In the practice, the solved EOPs by bundle adjustment are at the exposure time but the proposed position and attitude of body are at recorded time. So the traditional calibration equations should be represented to kinematic mode.

(8) $$r_{bc}^{b}(t_{c},t_{b})=R_{l}^{b}(t_{b})(r_{oc}^{l}\left(t_{c}\right)-r_{ob}^{l}\left(t_{b}\right))$$

(9) $$R_{c}^{b}(t_{c},t_{b})=R_{l}^{b}(t_{b})R_{c}^{l}(t_{c})$$

When the platform of MMS is in kinematic mode, the accuracy of 3D positioning is significantly affected by the exposure time delay. Therefore, the proposed calibration method has been developed to reduce the impact of the exposure time delay. In the following derivation, the magnitude of the exposure time delay is assumed to be a small unknown constant. Because it is small, the IMU rotation matrix is assumed fix during delay period. The derivation of the related equation is described below:

(10) $$r_{bc}^{b}\left(t_{c}\right)=R_{l}^{b}\left(r_{oc}^{l}\left(t_{c}\right)-r_{ob}^{l}\left(t_{c}\right)\right)=R_{l}^{b}(r_{oc}^{l}\left(t_{c}\right)-(r_{ob}^{l}\left(t_{b}\right)+r_{ob}^{l}\left(\Delta t\right))\;=R_{l}^{b}\left(r_{oc}^{l}\left(t_{c}\right)-r_{ob}^{l}\left(t_{b}\right)-{\dot{r}}_{ob}^{l}*\Delta t\right)=r_{bc}^{b}\left(t_{c},t_{b}\right)-v_{ob}^{b}*\Delta t\;R_{b}^{c}\left(t_{c}\right)=R_{l}^{c}\left(t_{c}\right)*R_{b}^{l}\left(t_{c}\right)=R_{l}^{c}\left(t_{c}\right)*R_{b}^{l}\left(t_{b}\right)*R_{b}^{l}\left(\Delta t\right)=R_{b}^{c}\left(t_{c},t_{b}\right)*R_{b}^{l}\left(\Delta t\right)\;\to R_{c}^{b}\left(t_{c}\right)=R_{l}^{b}\left(\Delta t\right)*R_{c}^{b}\left(t_{c},t_{b}\right)={\dot{R}}_{l}^{b}*\Delta t*R_{c}^{b}\left(t_{c},t_{b}\right)$$

where, “r”, “R” and “t” mean position vector, rotation matrix and time, respectively, and the superscript and subscript are the frame. We suppose the frames of INS/GNSS and MMS are overlaid, so “b” is the MMS body frame and also the INS/GNSS frame. The “c” and “l” are camera and local level frame, respectively.

The calibration process is carried out in the local level frame to avoid unnecessary systematic error sources due to coordinate transformation. The equation builds the relationship between the measurement and unknowns, including lever arm, boresight and exposure time delay (Δt). These are arranged and rewritten as shown below. The proposed method is implemented using the Least Square (LS) method. Before processing the LS, the rotation matrix function is re-written in Quaternions form, so the unknown items of boresight angle are [q0, q1, q2, q3]. In the coefficient matrix “A”, the coefficients of boresight angle are also differential by Taylor series. Therefore, the unknown are time delay [∆t], lever arms that have three elements including [x, y, z] in the body frame and the boresight angles that have four elements including [q0, q1, q2, q3]:

(11) $$\{\begin{array}{c} \Delta r_{bc}^{b}\left(t_{c},t_{b}\right)=r_{bc}^{b}\left(t_{c}\right)+v_{ob}^{b}*\Delta t\\ \Delta R_{c}^{b}\left(t_{c},t_{b}\right)=\Delta t*{\dot{R}}_{b}^{l}*R_{c}^{b}(t_{c})\\ \Delta L+V=AX\\ \Delta X={\left[r_{bc}^{b},R_{c}^{b},\Delta t\right]}_{8x1}^{T}\\ \Delta L={\left[r_{bc}^{b}\left(t_{c},t_{b}\right),R_{c}^{b}\left(t_{c},t_{b}\right)\right]}_{7x1}^{T} \end{array}$$

Generally speaking, the accuracy of the calibration procedure is dominated by the quality of the INS/GNSS POS data and the bundle adjustment results. This relationship also implicitly affects the performance of the MMS. The traditional calibration method does not calibrate the exposure time delay simultaneously. If the method is applied to calibrate an MMS operating in kinematic mode, the impact of the exposure time delay will propagate to the lever arm and boresight, respectively. The proposed method can avoid this problem and provide the best estimates of lever arm, boresight and exposure time delay at the same time. On another note, the distribution of the GCPs in the image and the quality of the INS/GNSS solutions are very important during the calibration procedure. After obtaining calibration parameters, the DG task can be performed seamlessly without GCPs as long as the spatial relationships of all the sensors within this MMS module remain fixed.

## 5. Data Processing Strategy

For the determination of the delay, lever arm and boresight parameters, the EOPs, including position and attitude of the images, must be solved using the bundle adjustment. However, some errors will occur during the image measurements due to imperfections of cameras during production. Thus the camera calibration must be performed. The objective of camera calibration is to analyze the interior orientation parameters (IOPs), such as the lens distortion, the focal length, and the principle point. If this is done carefully, systematic errors can be diminished during the image point measurements. Therefore, in order to process the system calibration above and check the ability of DG, the establishment of the camera control field and the ground control field must be done in this research. 

**Figure 11:**
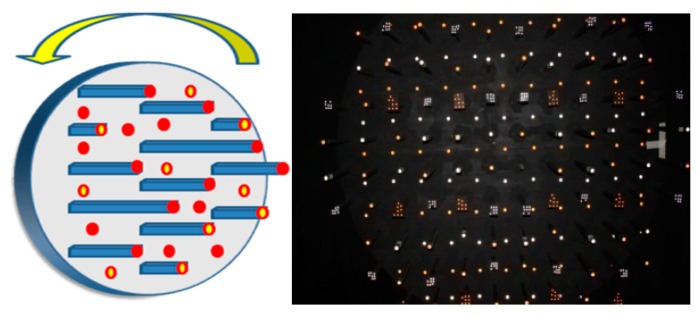
The camera control field.

A circular plane is set up for camera calibration. The diameter of this plane is 240 cm, and more than two hundred artificial landmarks are distributed evenly across it. Landmarks provided by Australis software are also included. Such design can be used to calibrate various cameras with different resolution and focal length process, as shown in Figure 11 [9].

**Figure 12:**
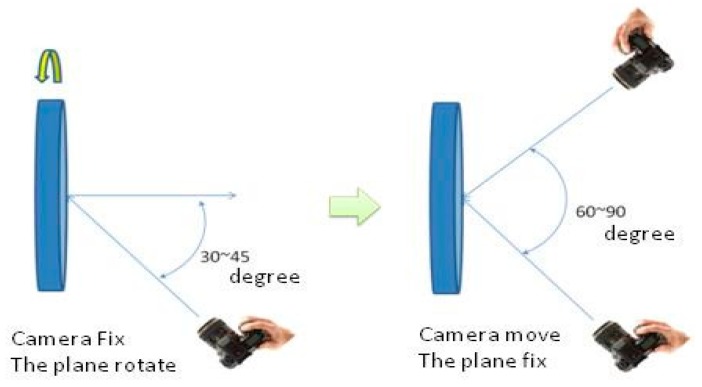
Relation between two situations.

Generally speaking, the amount of the images captured from multi-angle at the different locations could be restrained if the space of the camera control field is not enough. However, in the proposed field architecture, the relation of each landmark is fixed during the field rotation. That means its local coordinate system is also invariable. Each image can be shot at the same location but with different rotation angle. Compared to change the location of the shot, this design can overcome the restriction of the field space and provide sufficient reliability of the camera calibration, as shown in Figure 12. The camera control field is designed to acquire images with the best intersection geometry and avoid the high correlation between parameters. Thus, the calibration can be processed in the small space such as our control field (only 4 × 4 × 3 m^3^) with the best intersection geometry and the low correlation between parameters.

The analysis of IOPs such as the focal length, the principal point, and the lens distortion is the objective of this process. The bundle method with self-calibration is proposed to determine the interior parameters CCD cameras have applied. The equation is included in the bundle adjustment [16]:

(12) $${\text{x}}_{\text{a}}={\text{x}}_{\text{p}}-\text{c}\frac{{\text{r}}_{11}\left({\text{X}}_{\text{A}}-{\text{X}}_{\text{O}}\right)+{\text{r}}_{12}\left({\text{Y}}_{\text{A}}-{\text{Y}}_{\text{O}}\right)+{\text{r}}_{13}\left({\text{Z}}_{\text{A}}-{\text{Z}}_{\text{O}}\right)}{{\text{r}}_{31}\left({\text{X}}_{\text{A}}-{\text{X}}_{\text{O}}\right)+{\text{r}}_{32}\left({\text{Y}}_{\text{A}}-{\text{Y}}_{\text{O}}\right)+{\text{r}}_{33}\left({\text{Z}}_{\text{A}}-{\text{Z}}_{\text{O}}\right)}+\Delta \text{x}$$

(13) $${\text{y}}_{\text{a}}={\text{y}}_{\text{p}}-\text{c}\frac{{\text{r}}_{21}\left({\text{X}}_{\text{A}}-{\text{X}}_{\text{O}}\right)+{\text{r}}_{22}\left({\text{Y}}_{\text{A}}-{\text{Y}}_{\text{O}}\right)+{\text{r}}_{23}\left({\text{Z}}_{\text{A}}-{\text{Z}}_{\text{O}}\right)}{{\text{r}}_{31}\left({\text{X}}_{\text{A}}-{\text{X}}_{\text{O}}\right)+{\text{r}}_{32}\left({\text{Y}}_{\text{A}}-{\text{Y}}_{\text{O}}\right)+{\text{r}}_{33}\left({\text{Z}}_{\text{A}}-{\text{Z}}_{\text{O}}\right)}+\Delta \text{y}$$

where:

C: The focal length;
${\text{x}}_{\text{p}},{\text{y}}_{\text{p}}$: The principal points;
${\text{x}}_{\text{a}},{\text{y}}_{\text{a}}$: The coordinates of target A in camera frame;
${\text{r}}_{11}\sim {\text{r}}_{33}$: The rotation matrix;
${\text{X}}_{\text{A}}$,${\text{ Y}}_{\text{A}}$,${\text{ Z}}_{\text{A}}:$ The coordinates of target A in object frame;
Δx,Δy: The lens distortion.

This research adapts the commercial software, Australis [17], to solve for those parameters. It can process calibration automatically after the image is imported. A lens distortion model that includes seven parameters is enough for most kinds of cameras:

(14) $$\Delta \text{x}=\bar{\text{x}}+\left({\text{K}}_{1}{\text{r}}^{2}+{\text{K}}_{2}{\text{r}}^{4}+{\text{K}}_{3}{\text{r}}^{6}\right)\bar{\text{x}}+{\text{P}}_{1}\left({\text{r}}^{2}+2{\bar{\text{x}}}^{2}\right)+2{\text{P}}_{2}\text{xy}+{\text{b}}_{1}\text{x}+{\text{b}}_{2}\text{y}$$

(15) $$\Delta \text{y}=\bar{\text{y}}+\left({\text{K}}_{1}{\text{r}}^{2}+{\text{K}}_{2}{\text{r}}^{4}+{\text{K}}_{3}{\text{r}}^{6}\right)\bar{\text{y}}+{\text{P}}_{1}\left({\text{r}}^{2}+2{\bar{\text{y}}}^{2}\right)+\;2{\text{P}}_{1}\text{xy}$$

where: $\bar{\text{x}}=\left(\text{x}-{\text{x}}_{\text{p}}\right),\bar{\text{y}}=(\text{y}-{\text{y}}_{\text{p}}),\text{ r}=\sqrt{{\bar{\text{x}}}^{2}+{\bar{\text{y}}}^{2}}$

K_1_, K_2_ and K_3_: The radial lens distortion parameters;

P_1_ and P_2_: The decentric lens distortion parameters.

b_1_ and b_2_: The affine deformation parameters.

**Figure 13:**
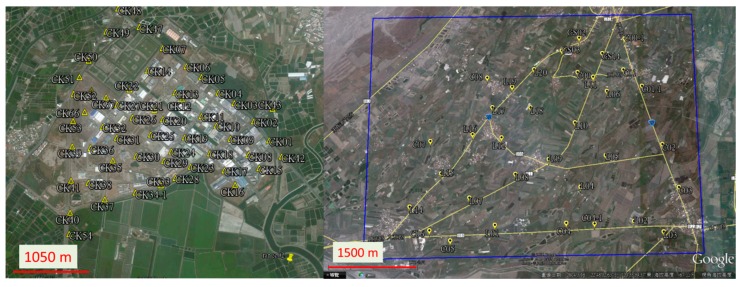
The distribution of GCPs in two control fields.

After obtaining proper IOPs, those parameters can be applied to enhance the accuracy of EOPs estimation of the bundle adjustment for system calibration and DG. For the determination of calibration parameters, the EOPs of each image need be known. They can be calculated using the bundle adjustment control field. So the two control fields are built for calibrating those systems applied in the study. Figure 13 illustrates the distribution of ground control points (GCPs) in two control fields which have been set up at distances of 400 and 800 m (Figure 13). The GCPs are accurately surveyed using differential-GNSS with carrier phase measurements and processed with network adjustment software. The standard deviation of GCPs is 3 mm.

The image acquisition for the system calibration process is performed via flying UAV photogrammetric over the ground control field. The measurements of the image points are measured first. Second, the Australis software is used to complete the bundle adjustment to get the EOPs of each image. After performing the interpolation of INS/GPS positioning and orientation solution (POS) at trigger time, the differences between the EOPs and interpolated POS are derived for further processing. The differences are used to calculate calibration parameters using the previously mentioned calibration algorithm. After obtaining calibration parameters, the DG task can be performed exactly without using any GCP. On the other hand, traditional photogrammetric processes can use INS/GPS POS and GCPs throughout the whole area of interest to assist the conventional bundle adjustments process [18].

**Figure 14:**
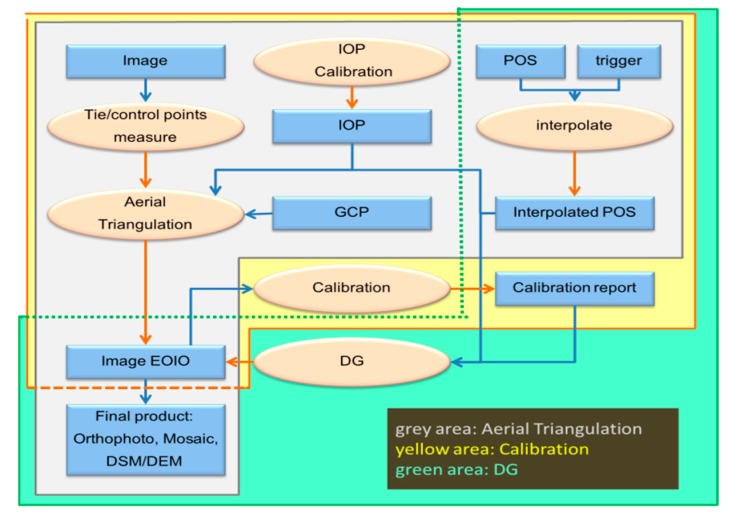
The process of INS/GPS POS assisted AT, system calibration and DG.

The grey, yellow and green scopes in Figure 14 illustrate the process of INS/GPS POS assisted AT, system calibration and DG, respectively. The INS/GPS POS helps the AT to execute AT after the three steps are finished, as tie-points and control points are measured, the IOPs of the cameras is calibrated and INS/GPS POS is interpolated. The EOPs of each image is obtained through AT, and the final products can be completed using programs like ortho-photo, Mosaic and DEM/DSM. INS/GPS POS assisted AT is included in the combination of calibration and DG processes, as shown in Figure 14. 

The calibration procedure requires the EOPs of each image and the interpolated INS/GPS POS solutions. Therefore, the calibration can be executed after completing AT, after which the calibration report is generated. The DG function can provide positioning of interesting points without using GCP with interpolated INS/GPS POS and calibration report. In fact, the final products of DG are the same as AT. However, the INS/GPS POS assisted AT has to implement dense GCPs throughout the whole area under analysis before taking pictures. On the other hand, the DG mode only requires a control field for calibration purposes which is not required for every mission once it has been performed; the payload remains fixed after the last calibration. 

**Figure 15:**
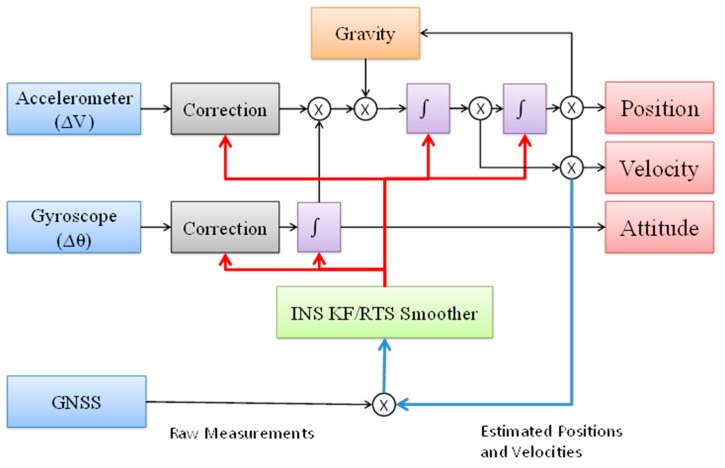
The TC integration scheme.

To avoid losing a lock on the GPS satellite due to the vibration of the UAV platform and certain maneuvers, this study applies the TC scheme to provide more robust POS solutions and overcome hardware limitations even when there may be frequent partial GPS outages to overcome. The TC scheme uses a single KF to integrate GPS and IMU measurements, as shown in Figure 15, which shows how the raw measurements are collected from the IMU and converted into position, velocity, and attitude measurements in the desired coordinate system using the INS mechanization algorithms. In the TC integration, the GPS pseudo range, delta range, and carrier phase measurements are processed directly in the INS KF [19]. The primary advantage of this integration is that raw GPS measurements can still be used to update the INS when fewer than four satellites are available. This is of special benefit in a complex environment, such as downtown areas where the reception of the satellite signals is difficult due to obstruction. Also, in cases when carrier phase GPS measurements are used, the IMU measurements can be used to reduce ambiguity in the resolution algorithm.

Post-mission processing, when compared to real-time filtering, has the advantage of using the data of the whole mission for estimating the trajectory. It is impossible using the whole data to filtering on real-time because only part of data is available except the last. After filtering is used in the first step, an optimal smoothing method, such as the Rauch-Tung-Striebel (RTS) backward smoother, can be applied [4]. This uses filtered results and their covariance as a first approximation which is then improved by using additional data that was not used in the filtering process. Depending on the type of data used, the improvement obtained by optimal smoothing can be considerable [20].

**Figure 16:**
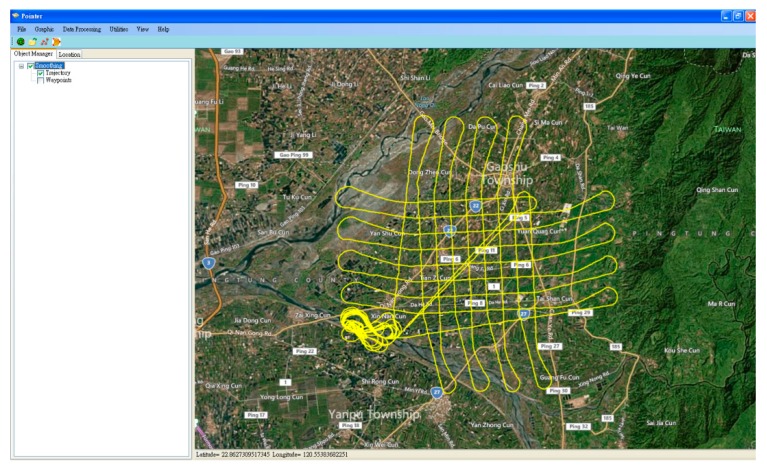
POS software.

For a geo-referencing process which puts POS stamps on images and a measurement process that obtains three-dimensional coordinates of all important features and stores them in a Geographic Information System (GIS) database, only post-mission processing can be implemented due to the complexity of the task [21]. Therefore, most commercially available DG systems operate in real-time only for data acquisition and conduct most of the data processing and analysis in post-mission mode. Figure 16 illustrates the POS software (Pointer. POS) developed in this study, which includes the GNSS processing engine, INS mechanizations in different navigation frames, as well as the optimal estimation engine, which can perform optimal fusion in LC, TC and Hybrid Tightly Coupled (HTC) schemes. 

After processing POS and bundle adjustment solutions using measurements acquired over control fields, calibration and performance verification can be achieved. First, the position and attitude of POS must be converted to [x, y, z] and the normalized quaternions form for the further processing. The smoothed POS solutions are interpolated by linear interpolation at trigger time. To keep the coordinates consistent, the POS coordinates need to be converted to coordinates of interest. This can be performed through series of transformation methods [22]. 

**Figure 17:**
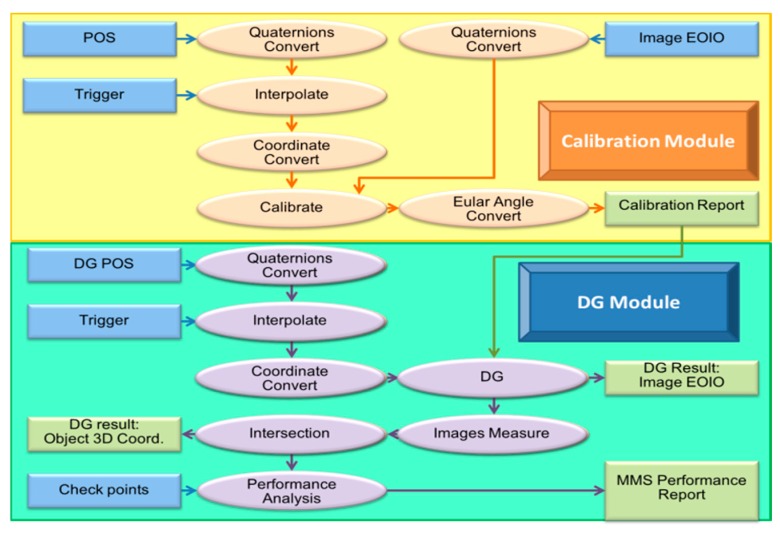
Data processing procedure.

The DG procedure is done by using smoothed POS solutions at trigger time and a calibration report to obtain IOPs and EOPs of each image. The three-dimensional coordinates of points of interest can be solved by conventional photogrammetric technology such as collinearity equation and intersection. The statistical analysis of MMS performance is estimated by check points and then output to the MMS performance report. Figure 17 illustrates the data processing procedure adopted in this study.

## 6. Results and Discussion

**Figure 18:**
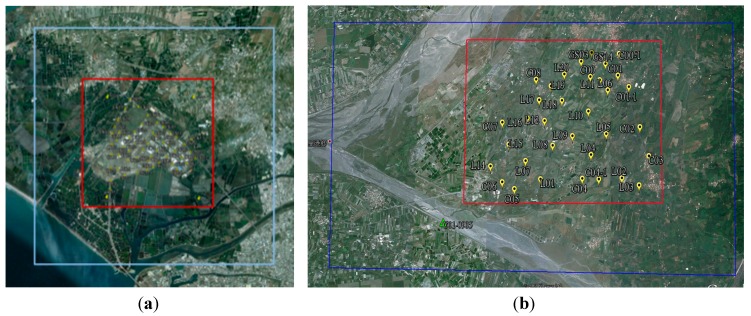
The scopes of the two tests.

To validate the impact of flight height on DG performance, a field test was conducted in the fall of 2011 at the first control field. The DG payload used in this scenario was the previous version and the flight altitudes set for aerial photography were set to 300 and 600 m above ground. The scope of the test zone is 3 km × 3 km, which covers the first control field, as shown in Figure 18a with the red square. The blue region illustrates the fly zone approved for this test. In addition, to compare the performance of previous and new versions of DG modules, the second test was conducted in the fall of 2013 at a second control field. The tested IMU was the ADIS16488 IMU and the flight altitude set for aerial photography was set to 300 and 600 m above ground in this test. The scope of the test zone is 3 km × 3 km, which covers the second control field shown in the red square in Figure 18b. The blue region illustrates the fly zone approved for this test.

**Figure 19:**
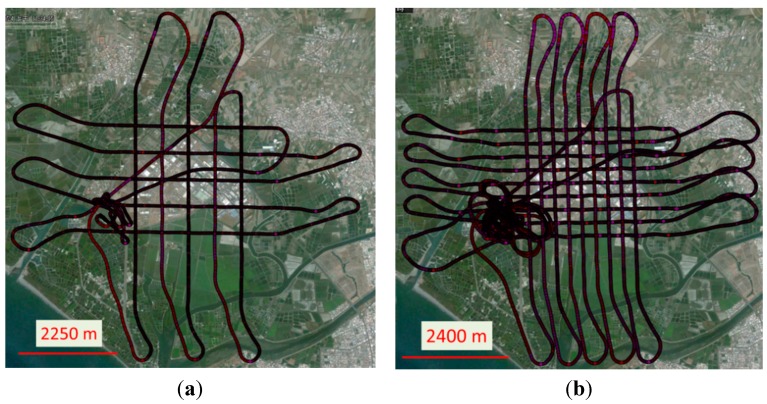
The trajectories of the first test flight. (**a**) UAV-MMQG-600; (**b**) UAV-MMQG-300.

**Figure 20:**
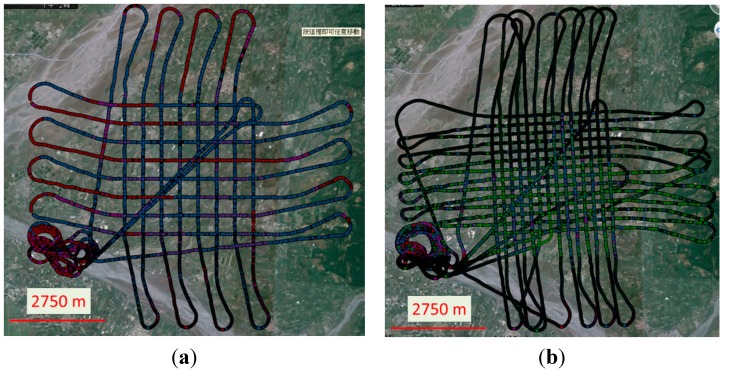
The trajectories of the second test flight. (**a**) UAV-ADIS-600; (**b**) UAV-ADIS-300.

Due to the effect of side winds, the attitude of UAV, the transversal and longitudinal overlapping were increased to 80% and 40% respectively to insure that the coverage of the stereo pair would overlap completely during the test flight. Although more images will have to be processed, this method guarantees complete coverage by the stereo pair. Figure 19 illustrates the flight trajectories of the first test at flight heights of 600 and 300 m which calls UAV-MMQG. Figure 20 depicts the trajectory of the second test which calls UAV-ADIS. The ground sample distances (GSD) of 600 m and 300 m of flight heights are about 20 cm and 10 cm.

### 6.1. Calibration Results

**Figure 21:**
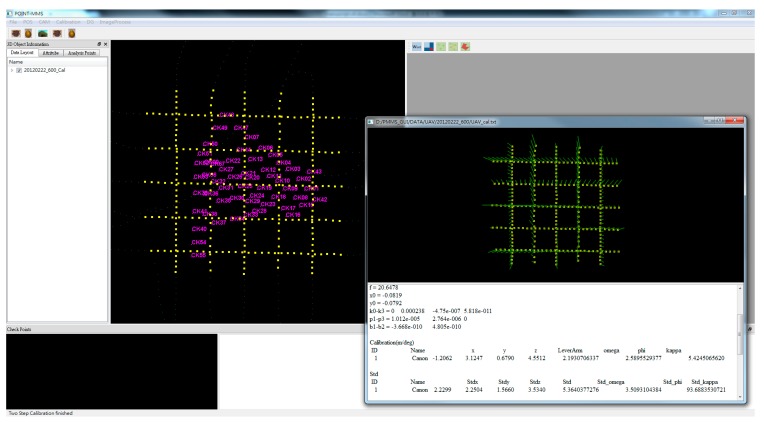
The calibration operation of the program.

Traditional and proposed calibration procedures are implemented in this study to estimate calibration parameters of each camera for further study. The proposed software, as shown in Figure 21, was developed using Visual Studio 2008 C++, QT, OpenCV and OpenGL for system calibration and DG verification. 

The EOPs of the images are calculated first with Australis software through the bundle adjustment by measuring the image points when the flight mission has been completed. Then the trajectories of INS/GPS integrated POS are obtained through the use of TC schemes with the RTS smoother. The interpolation of INS/GPS smoothed POS solutions at the image exposure time is then performed. The lever arm and boresight angle for each epoch are applied by comparing the differences of the position and the attitude between the exterior orientation parameters and the interpolated INS/GPS solutions. The proposed software solves these calibration parameters using the methods described, and generates a calibration report, as shown in Table 1.

**Figure 22:**
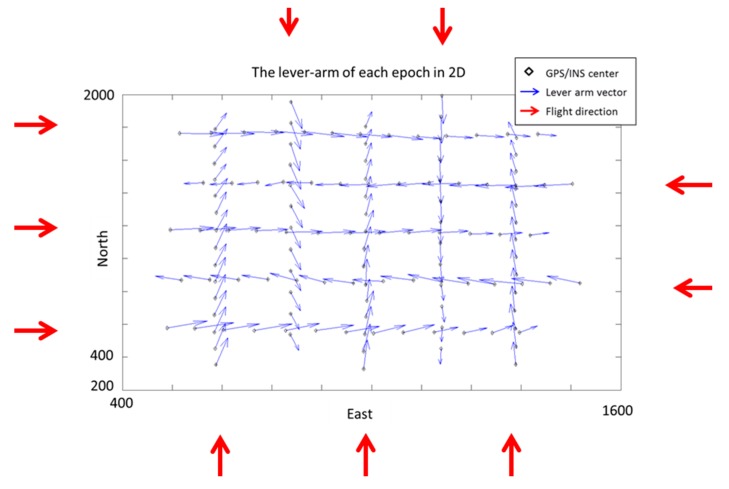
The lever-arm of each epoch.

The center of POS and the camera is roughly overlaid along the x and y axis when they are assembled in the payload frame. As shown in Table 1, the relative accuracy of the proposed method is better than that of the traditional calibration method. The most probable values and standard deviation of DG modules with the 600 and 300 m flight heights are compared based on those calibration methods. As illustrated in Table 2, the standard deviation and the most probable values of the 300 meter flight height scenario is much better than those of the 600 m flight height scenario for both calibration methods. This finding illustrates the fact that the calibration flight test should be conducted at height of 200–300 m for the UAV used in this study. In addition, the relative accuracy and the most probable values for the new DG module illustrate results superior to those of the previous DG module, showing that the calibration results can be improved significantly with a better POS module. In addition, the accuracy of the proposed calibration method is superior to the traditional calibration method because the bias of exposure time delay contaminates lever-am and boresight parameters in the traditional calibration method. Figure 22 illustrates the impact of exposure time delay on lever arm parameters at each epoch. Because the center of IMU is designed on overlaying the center of camera, the lever-arm should be near zero in the level direction. The delay leads to a bias which depends on the velocity of the platform in the forward direction.

**Table 1:** The results of two calibration methods.

		(s)	Lever-Arm (m)			Boresight (deg)		
		Delay	X	Y	Z	Omega	Phi	Kappa
UAV MMQG 600	Traditional Calibration							
	most probable value		−1.2062	3.1247	0.6790	3.79797404	5.20251129	−2.24883285
	Standard deviation		2.0572	1.1891	2.7717	3.69862986	3.49680553	5.86546832
	Proposed Calibration							
	most probable value	−0.1072	−1.1205	−0.001	1.0557	4.61012742	5.21063752	−1.79112843
	Standard deviation	0.0036	0.0714	0.1317	0.0858	0.54718413	0.37337511	0.22203555
UAV MMQG 300	Traditional Calibration							
	most probable value		0.0141	3.8105	0.1513	4.40651630	0.51021908	−0.00873761
	Standard deviation		0.4595	1.1863	1.1839	4.37609122	5.47624647	3.77899241
	Proposed Calibration							
	most probable value	−0.1272	−0.0548	0.1523	0.3580	4.38480491	0.53859868	0.25962901
	Standard deviation	0.0043	0.0767	0.1125	0.0771	1.53376932	1.63520332	2.09804173
UAV ADIS 600	Traditional Calibration							
	most probable value		−2.0806	6.1863	1.3527	1.26444091	0.500277771	−0.193128818
	Standard deviation		2.2299	2.2504	1.5660	2.61737788	1.99383288	3.03577883
	Proposed Calibration							
	most probable value	−0.2272	−2.1138	−0.2578	1.8353	1.34584366	0.51119238	−0.28419414
	Standard deviation	0.0050	0.0804	0.1293	0.0811	0.15690974	0.14208760	0.14200760
UAV ADIS 300	Traditional Calibration							
	most probable value		−0.8034	3.3460	−0.0718	2.464419253	4.499372566	−1.291911667
	Standard deviation		0.6815	0.7948	0.7347	4.31193476	1.05151768	1.70347184
	Proposed Calibration							
	most probable value	−0.1394	−0.6251	−0.3909	0.1992	2.37524174	4.51749719	3.28462927
	Standard deviation	0.0026	0.0425	0.0703	0.0430	0.99552547	0.07104126	0.06245981

**Table 2:** The statistical analysis of DG based on traditional calibration method.

Traditional Calibration						Proposed Calibration				
(m)	E	N	U	2D	3D	E	N	U	2D	3D
UAV-MMQG-600(48)										
AVG	0.0560	−2.8740	−0.3470	2.8745	2.8954	−1.5370	−4.6440	1.1730	4.8917	5.0304
STD	11.2300	8.3610	15.4680	14.0007	20.8633	10.4340	7.9770	13.8660	13.1340	19.0989
RMS	11.1240	8.7660	15.3250	14.1628	20.8672	10.4390	9.1590	13.7710	13.8874	19.5576
UAV-MMQG-300(51)										
AVG	−0.5090	0.0550	−3.3960	0.5120	3.4344	0.1880	−1.6330	−1.2860	1.6438	2.0871
STD	8.9410	6.5850	14.9560	11.1042	18.6276	8.2970	6.1900	11.4760	10.3516	15.4549
RMS	8.8680	6.5210	15.1930	11.0075	18.7615	8.2190	6.3440	11.4380	10.3826	15.4475
UAV-ADIS-600(32)										
AVG	−0.1040	−0.9900	−1.2560	0.9954	1.6026	0.0480	0.9050	−1.3990	0.9063	1.6669
STD	5.9380	7.3070	11.2860	9.4155	14.6978	6.0310	6.9060	10.0350	9.1687	13.5929
RMS	5.8440	7.2490	11.1790	9.3113	14.5489	5.9370	6.8680	9.9750	9.0784	13.4877
UAV-ADIS-300(24)										
AVG	0.4880	−1.3150	−0.0100	1.4026	1.4027	−0.4850	−0.3880	−1.4470	0.6211	1.5747
STD	4.6590	5.5380	5.2160	7.2371	8.9209	3.8810	4.5650	5.2340	5.9918	7.9559
RMS	4.5870	5.5780	5.1060	7.2218	8.8445	3.8300	4.4850	5.3240	5.8978	7.9454

### 6.2. Verification of the DG Capability of the Proposed UAV Photogrammetric Platform

**Figure 23:**
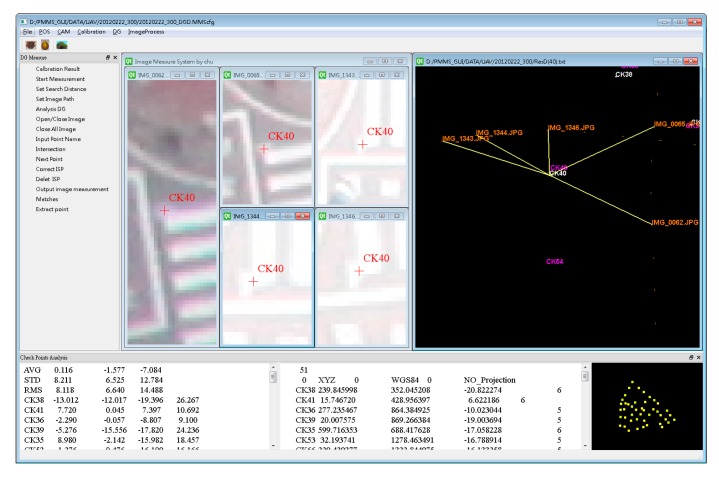
The DG program.

The software developed in this study can also perform the DG verification using a collinearity equation and intersection to calculate the coordinates of the check point, as shown in Figure 23, which presents the relevant information, including the coordinates of the control points, POS, calibration report and trigger file—which have been imported into the software which calculates the EOPs for each image using the DG function. Users can perform image point measurements of the check points which appear in the different images. The results of the space intersection of check points are obtained from these images, after which their coordinates, derived through GCP free mode, are then compared with the already-known coordinates. The reference coordinates of the check points are obtained through the precise control survey with GNSS RTK technology and network adjustment. Therefore, the DG coordinates of those check points can then be compared with their reference coordinates. 

**Figure 24:**
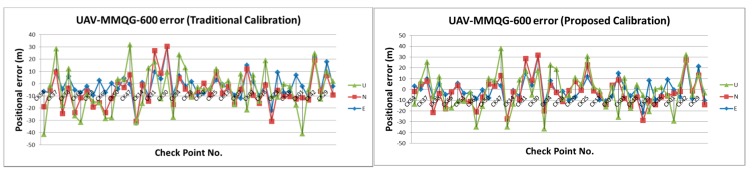
DG error based on MMQG with 600 m.

**Figure 25:**
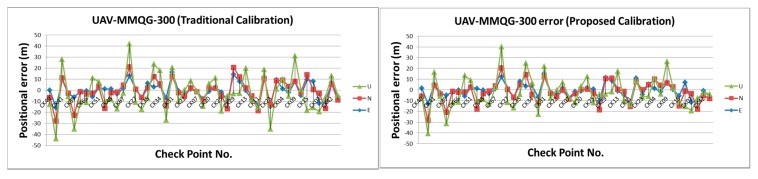
DG error based on MMQG with 300 m.

**Figure 26:**
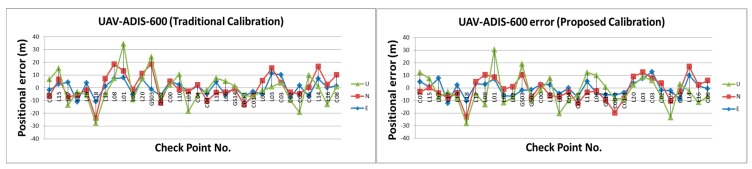
DG error based on ADIS 16488 with 600 m.

**Figure 27:**
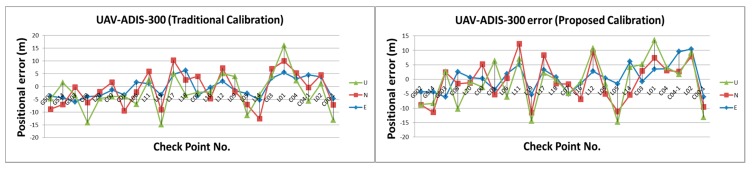
DG error based on ADIS 16488 with 300 m.

**Figure 28:**
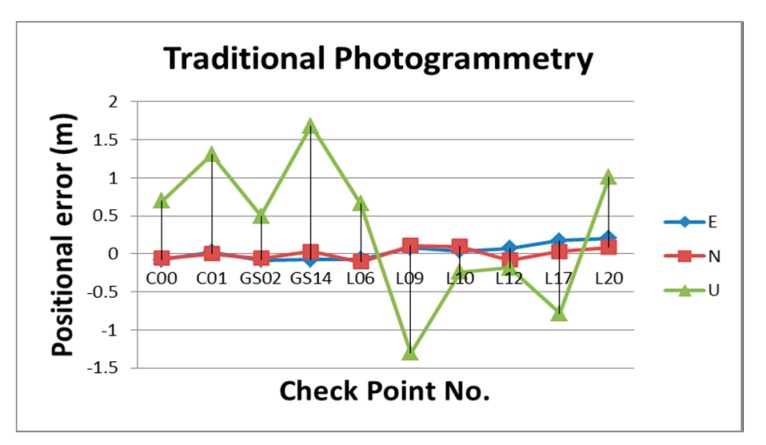
The positional errors of traditional photogrammetry based on ADIS 16488 with 300 m.

Table 2 illustrates the statistical analysis of DG based on different flights height, DG modules and calibration methods. Figure 24, Figure 25, Figure 26 and Figure 27 illustrate the DG performance for the scenarios with those check points. It can clearly be seen that the newly developed DG payload is significantly better than the previous version of the DG payload. The horizontal positioning accuracy of new DG is best at about 5.8 m in 2D and 7.9 m in 3D. On the other hand, Figure 28 and Table 3 illustrate the positional errors with traditional photogrammetry using data acquired by ADIS 16488 with 300 m flying height. The following are several comparisons of three factors which are hardware, flight height and calibration method.

**Table 3:** The statistical analysis of traditional photogrammetry based on ADIS 16488 with 300 m.

meter	E	N	U
AVG	0.028	0.005	0.333
AVG	0.105	0.079	0.943
RMS	0.104	0.075	0.954

Table 4 and Table 5 illustrate the improvement rates analysis for these scenarios. The first scenario is the relationship of the low cost POS to the flight height. The accuracy is based on MMQG, with the 300 m flight improved by about 28.7% and 11.2% in term of 2D and 3D absolute positional errors, respectively, as compared with the 600 meter flight height using the traditional calibration method. At the same time, the accuracy of the ADIS 16488 also improves by 28.9% and 64.5% in terms of 2D and 3D. The statistical numbers of MMQG and ADIS16488 IMU improve by 33.8%, 26.6% and 53.9%, 69.8% using the proposed method.

**Table 4:** The improvement rate of DG accuracy based on different flight and DG modules.

%			MMQG-300	ADIS16488-300
Traditional Calibration	MMQG-600	2D	28.67	96.11
		3D	11.22	135.93
	MMQG-300	2D	0.00	52.42
		3D		112.12
	ADIS16488-600	2D	−18.22	28.93
		3D	−28.95	64.50
Proposed Calibration	MMQG-600	2D	33.76	135.47
		3D	26.61	146.15
	MMQG-300	2D	0.00	76.04
		3D		94.42
	ADIS16488-600	2D	−14.37	53.93
		3D	−14.53	69.76

The second scenario compares the performance of two DG modules. Comparing the two results when using MMQG-600 and ADIS16488-300, the results of ADIS16488 IMU are superior to those of MMQG. It improves by 96.1% and 135.9% in terms of 2D and 3D, respectively, for absolute positional errors using the traditional calibration method, and improves by 135.5% for 2D and 146.2% for 3D in terms of absolute positional errors using the proposed method. 

The third scenario is the relationship between two IMUs. The improvements of proposed IMU are about 52.4% and 112.1% with 300 meter flight height and 76.0% and 94.4% with 600 meter flight height compared to MMQG.

The last analysis is the improvement rate based on the new calibration method. The proposed method has proven effective in all scenarios to which it has been applied in this study. Based on MMGQ, it improves 2.0% and 6.7% in terms of 2D and 3D, respectively, in regards to absolute positional errors for the 600 m flight and 6.0% and 21.5% in terms of 2D and 3D, respectively, for absolute positional errors for the 300 m flight. The DG accuracy levels provided by the proposed DG modules with the proposed method reach 2.6% for 2D and 7.5% for 3D in absolute positional errors for the 600 m flight and 22.5% and 11.3% for the 300 m flight.

**Table 5:** The improvement rate of DG accuracy with the proposed method.

%	Proposed Calibration	
	2D	3D
MMQG-600	1.98	6.70
MMQG-300	6.02	21.45
ADIS16488-600	2.62	7.52
ADIS16488-300	22.45	11.32

The approximate error budgets of the proposed tactical grade DG module for flight heights of 600 and 300 m are given in Table 6. (Table 3 also looked at how the primary DG positional error sources are related to the quality of the gyroscopes used within the IMU.) The proposed DG module improves the kinematics positioning accuracy of trajectory to within less than 1 m by using single frequency carrier phase measurements. In addition, the remaining positional error sources can be mitigated by replacing the current IMU with superior gyroscopes.

**Table 6:** Error budgets of the new DG system.

	ADIS 16488 with 600 Flight Height		ADIS 16488 with 300 Flight Height	
Error source	Magnitude	Impact on (DG Error)	Magnitude	Impact on (DG Error)
INS/GNSS Positional error	0.1–0.2 m	0.1–0.2 m	0.1–0.2 m	0.1–0.2 m
INS/GNSS Orientation error	0.15–0.25 degree	1.6–2.5 m	0.15–0.25 degree	1.6–2.5 m
Calibration error -Boresight -Lever-arm	0.15–0.25 degree 0.1–0.2 m	1.6–2.5 m 0.1–0.2 m	0.15–0.25 degree 0.1–0.2 m	0.8–1.3 m 0.1–0.2 m
Synchronization error -Position -Orientation	1–2 ms	120 km/h fly speed0.036–0.072 m0.3–0.6 m	1–2 ms	120 km/h fly speed 0.036–0.072 m 0.3–0.6 m

The primary contribution of this study is the implementation of a UAV based photogrammetric platform with DG ability and the verification of its performance in terms of DG accuracy for various situations using a low cost tactical grade IMU. In addition, the preliminary results indicate that the DG accuracy in GCP free mode can meet the requirements for rapid disaster mapping and relief applications.

The total cost of the proposed POS module is below 2000 US dollars, making it suitable for rapid disaster relief deployment to provide near real-time geo-referenced spatial information. The data processing time for the DG module, including POS solution generalization, interpolation, EOP generation, and feature point measurements, is less than one hour.

## 7. Conclusions

This study develops a long endurance DG based fixed-wing UAV photogrammetric platform in which a low cost tactical grade integrated Positioning and Orientation System (POS) is developed. In addition, a novel kinematic calibration method including lever arms, boresight angles and camera shutter delay is proposed and compared with traditional calibration method. Furthermore, the performance of DG is also analyzed based on the two methods with different flights and two DG modules. The results presented in this study indicate that the accuracy of DG can be significantly improved by lower flight heights and hardware with superior specifications. The proposed method improves the accuracy of DG by about 10%. 

The preliminary results show that horizontal DG positioning accuracies in two-dimension (2D) are around 8 m at a flight height of 600 m with the newly designed tactical grade integrated Positioning and Orientation System (POS). The positioning accuracy in three-dimensions (3D) is less than 12 m. Such accuracy is good for near real-time disaster relief. 

The DG ready function of the proposed platform guarantees mapping and positioning capability even in GCP free environments, which is very important for rapid urgent response for disaster relief. Generally speaking, the data processing time for the DG module, including POS solution generalization, interpolation, Exterior Orientation Parameters (EOP) generation, and feature point measurements, is less than one hour.
